# Mobile Health App for Adults with Persisting Postconcussion Symptoms: Development and Usability Study

**DOI:** 10.2196/75323

**Published:** 2025-11-04

**Authors:** Gøril Storvig, Anker Stubberud, Johanne Rauwenhoff, Liv Marie Rønhovde, Martijn Smits, Simen Berg Saksvik, Toril Skandsen, Erling Tronvik, Alexander Olsen

**Affiliations:** 1 Department of Psychology, Norwegian University of Science and Technology, Trondheim, 7491, Norway, +47 73591960; 2 Clinic of Rehabilitation, St Olav's University Hospital, Trondheim, Norway; 3 Norwegian Center for Headache Research, Trondheim, Norway; 4 Department of Neuromedicine and Movement Science, Norwegian University of Science and Technology, Trondheim, Norway; 5 Department of Research and Development, St Olav's University Hospital, Trondheim, Norway; 6 Department of Neurology, St Olav's University Hospital, Trondheim, Norway

**Keywords:** mTBI, mild traumatic brain injury, concussion, postconcussion syndrome, postconcussive syndrome, postconcussive symptoms, persistent postconcussion symptoms, mobile health, symptom tracking, symptom monitoring, persisting symptoms after concussion, PSaC

## Abstract

**Background:**

Diagnostics, treatment, and research of persisting postconcussion symptoms are challenging. Assessing symptoms is essential, but currently implemented methods only allow for retrospective reporting of symptoms. A mobile health (mHealth) symptom mapping app for adults with persisting postconcussion symptoms may be an accessible and cost-efficient alternative.

**Objective:**

This study aimed to develop a research-based mobile app for symptom mapping for adults with persisting postconcussion symptoms and investigate its usability, feasibility, and safety.

**Methods:**

This was a mixed method development and usability study consisting of three iterative cycles, each including (1) app design and programming, (2) app usability evaluation by the user group, and (3) app review by the clinician group. The outcomes were the mHealth App Usability Questionnaire and Mobile App Rating Scale scores, the number of days with logged symptom data during a home-testing period, and descriptions of adverse events throughout the study period. Semistructured interviews were conducted to explore the user group’s experiences further.

**Results:**

Twenty-three adults with persisting postconcussion symptoms (median age 52, IQR 34-59 years; 70% female) were included in the user group. Six clinicians (median age 53, IQR 35-60 years), including 3 (50%) females, with a mean of 13 (SD 7) years of experience working with individuals with persisting postconcussion symptoms, were included in the clinician group. The app received a mean score of 5 (SD 1.1) on the mHealth App Usability Questionnaire (7-point Likert scale) from the user group and 4.1 (SD 0.4) on the Mobile App Rating Scale (5-point Likert Scale) from the clinician group. During the 28-day home-testing period, the adherence rate among the participants in the user group was 89% (IQR 78‐96), and two adverse events related to increased symptom awareness were registered. Three themes were created through reflexive thematic analysis of the qualitative data: (1) Visualizing the invisible—Enabling reflection and insight; (2) Personalized yet simple—Balancing relevance and usefulness; and (3) More than just a number—The complexity behind the symptom scores.

**Conclusions:**

We developed a research-based symptom mapping app for people with persisting postconcussion symptoms. The app received high usability ratings from both the user and clinician groups. The app is a feasible alternative to traditional symptom mapping methods, and it is safe to use for its intended purpose.

## Introduction

Mild traumatic brain injury (mTBI) can be defined as a trauma to the head accompanied by acute signs of reduced cerebral functioning, such as brief loss of consciousness, confusion, or amnesia for the event, but often with no neuroradiological findings [1]. Common causes of civilian mTBI are falls, bicycle accidents, sports, motor vehicle accidents, being struck by objects, and violence [2]. People with mTBI can experience symptoms such as headaches, dizziness, nausea, fatigue, light sensitivity, sound sensitivity, trouble concentrating, attention problems, sadness, irritability, anxiety, depression, and sleep problems [3,4]. Most people recover within a few weeks after the injury, but a considerable proportion develop persisting postconcussion symptoms (PPCS) where symptoms persist for more than 3 months [5]. The reported prevalence of PPCS differs between contexts and study procedures, and a recent review found an estimated prevalence between 18.3% and 31.3%, depending on the applied definition of PPCS [6,7].

Despite the high prevalence, the etiology of PPCS is largely unknown and is an ongoing topic of debate among experts in the field [4,8]. Several factors, including pre, peri, and postinjury factors, are believed to influence or contribute to PPCS [9], and a biopsychosocial framework is often applied to understand these intricate processes [10,11]. PPCS is associated with reduced health-related quality of life [12], reduced return-to-work rate [13-15], and reduced participation in social life [16]. Regardless of the pathophysiological mechanisms involved, PPCS is a substantial health problem affecting the lives of many people.

Despite the high burden, we lack consensus on the diagnosis and classification of PPCS [4,17]. No single test can confirm PPCS; the diagnosis is established after careful evaluation of the medical history and self-reported symptoms [18,19]. Differential diagnosis can be particularly challenging, as similar symptoms to PPCS have also been reported in the general population [20].

Self-reporting of symptoms is essential in diagnosing and treating PPCS and is an important data source in research aiming to expand our understanding of the condition and evaluate treatment effects. Existing validated and widely used instruments for assessment of postconcussion symptoms, such as the Rivermead Postconcussion Symptom Questionnaire (RPQ) and the British Columbia Postconcussion Symptom Inventory, only allow for retrospective reporting, using a timescale of days or weeks [7,21,22]. While retrospective symptom assessment has pragmatic advantages, it may introduce recall bias, where the person reporting their symptoms fails to accurately recall the symptoms’ occurrence or intensity [23]. This can potentially lead to an incomplete representation of the person’s experienced symptoms, complicating the diagnosis and treatment of PPCS, as well as impeding research developments. Prospective symptom assessment, by comparison, has the benefit of the person reporting closer in time to symptom occurrence and may provide a more nuanced representation of symptom fluctuation and reduce the risk of recall bias [24].

An mHealth app for mapping PPCS is a highly warranted tool. It allows symptom registration in real time, capturing day-to-day fluctuations, and has the potential to support people with PPCS to take ownership of and manage their condition [25], support the clinician in diagnosing and evaluating treatment effects, and provide prospectively collected data for clinical trials. Furthermore, a mobile app is easily distributed, making it an accessible and cost-efficient alternative to traditional methods [26]. Therefore, we aimed to develop a mobile app for symptom mapping for people with PPCS and investigate its usability, feasibility, and safety.

## Methods

### Study Design and Participants

This was a mixed method iterative development and usability study carried out at St. Olavs University Hospital from December 25, 2022, to March 5, 2024. The mobile app was developed throughout the study based on feedback from people with PPCS (user group) and clinicians with expertise in PPCS (clinician group) at 3 different time points. The primary objective was to assess the app’s usability, and the secondary objectives were to assess feasibility and safety.

The study participants in the user group were recruited from an outpatient rehabilitation clinic, through announcements on the hospital’s official website, the Norwegian Center for Headache Research website, and user organizations and their social media. Inclusion criteria were: (1) age 18 years or older, (2) mTBI as defined by having sustained a head injury with observed or self-reported alteration of consciousness, amnesia, or other relevant acute symptoms, such as drowsiness, (3) PPCS according to the *International Classification of Diseases* research criteria operationalized as reporting 3 or more symptoms (including headache) on a moderate or greater intensity level on the RPQ, (4) posttraumatic headache according to the *International Classification of Headache Disorders 3rd edition*, (5) proficiency in the Norwegian language, and (6) signed written informed consent. Exclusion criteria were (1) severe psychiatric or somatic disease or other patient-related factors that would provide obvious challenges for adhering to the study protocol, including using the app, (2) having no access to an iOS (Apple Inc) or Android smartphone (Google LLC), or (3) having less than 3 months of smartphone experience. An experienced research nurse screened participants for eligibility by collecting relevant medical information and symptom history directly from the participants.

The study participants in the clinician group were identified based on their expertise in the field of PPCS and were recruited through convenience sampling using professional networks of the authors at Norwegian universities, hospitals, and clinics, aiming to include clinicians from diverse multidisciplinary backgrounds.

### Study Measures

The primary outcome was app usability, which was measured using the mHealth App Usability Questionnaire (MAUQ) and Mobile App Rating Scale (MARS). The MAUQ consists of 21 statements divided into 3 subsections: Ease of use and satisfaction, Interface and satisfaction, and Usefulness. The questionnaire has a 7-point Likert scale from 1 (strongly disagree) to 7 (strongly agree). The MARS is an app quality rating tool that assesses app quality on 4 dimensions: Engagement, Functionality, Aesthetics, and Information quality. The questionnaire uses a 5-point Likert scale, ranging from 1 (Inadequate) to 5 (Excellent). The total mean score describes the app’s overall quality, while mean subscale scores within the dimensions can be used to describe specific strengths and weaknesses. Professional translators translated the MAUQ and the MARS into Norwegian using a forward-backward translation method. Experts in the field of mTBI performed the harmonization of the translations.

The RPQ was completed as part of the screening and inclusion of study participants in the user group, as well as before and after the home testing period. The RPQ contains 16 items representing different symptoms and operates on a 5-point Likert scale from 0 (no experience at all) to 4 (severe problem). When summing up the scores, responses of 1 (no more of a problem than before) were recoded to 0, with a total score range of 0‐64. The Norwegian version of the RPQ has been translated from the original English version by the Collaborative European NeuroTrauma Effectiveness Research in Traumatic Brain Injury (CENTER-TBI) Consortium [27] and has been validated [28].

The secondary outcomes were feasibility and safety. To assess feasibility, we recorded the percentage of days with logged symptom data in the user group during the home-testing period of 28 days, referred to as adherence. For participants experiencing technical issues in the app hindering them from using it, the percentage was calculated as days with logged symptom data divided by the number of days it was possible to use the app. The app safety was assessed with the frequency of adverse events (AE), serious adverse events, and unexpected serious adverse events throughout the whole study period.

To further explore the app’s usability, feasibility, and safety, semistructured interviews were conducted with all participants in the user group as part of each iteration.

### Study Procedure

The study consisted of 3 iterative cycles. Each iteration included (1) app design and programming, (2) usability evaluation by the user group, and (3) review by the clinician group. During usability evaluation (2), the user group completed the MAUQ and conducted a semistructured interview, while the clinician group filled out the MARS during the app quality review (3). Before filling out the questionnaires, both the user and clinician groups had the opportunity to explore the app independently. Thereafter, they were instructed to perform specific tasks, typically describing an overarching goal, that is, “perform a registration of the symptoms you are experiencing today.”

**Figure 1.**
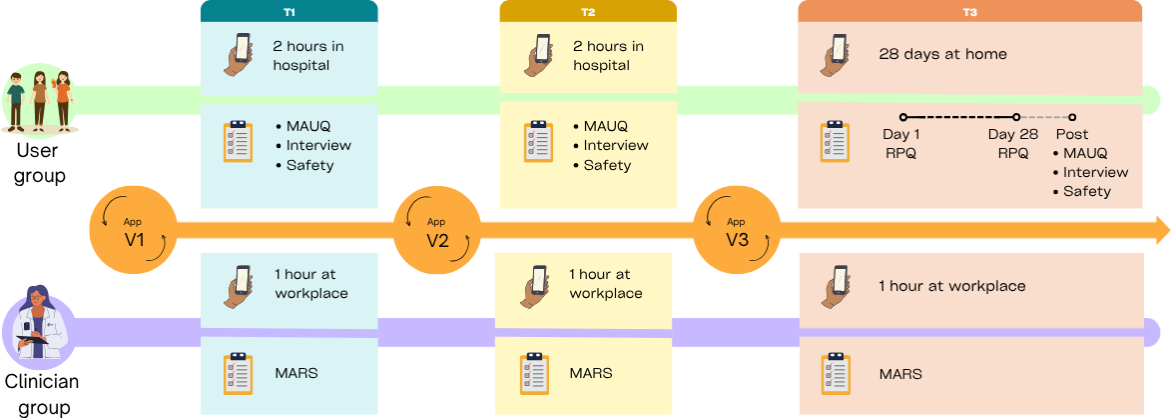
Illustration of the study procedure. The study participants evaluated the different versions of the app (v1, v2, and v3) as part of every iteration. In the first two iterations, the user group evaluated v1 and v2 during 2-hour sessions at the hospital, completing the mHealth App Usability Questionnaire, an interview, and a safety assessment. In the third iteration, they used v3 at home for 28 days, completed the Rivermead postconcussion symptom questionnaire on the first and last day, and completed the mHealth App Usability Questionnaire, an interview, and a safety assessment within 2 weeks after the last day. The clinician group evaluated v1, v2, and v3 in 1-hour sessions at their workplace, completing the Mobile App Rating Scale during all iterations. MARS: Mobile App Rating Scale; MAUQ: mHealth App Usability Questionnaire; RPQ: Rivermead Postconcussion Symptom Questionnaire.

The study participants’ immediate reactions and issues that emerged during the usability testing were also documented. The feedback from the user and clinician groups during usability testing was used to develop the next app version for the following iteration. In the first 2 iterations, the participants in the user group attended 2-hour individual sessions in a hospital setting to evaluate the app’s usability. While in the third iteration, they used the app for 28 days at home (Figure 1).

During the home testing, the user group was asked to use the app to register their symptoms daily. The app was preconfigured to send a notification every evening at 8 PM with the message “Did you experience symptoms today?*”* The study participants could adjust the notification time or disable it in the settings. They were able to register symptoms experienced within the last 48 hours but were not able to edit or add symptoms beyond that timeframe. Study personnel made 3 planned phone calls (approximately once a week) to assess any technical issues with the app. Additional phone calls were scheduled for further troubleshooting if technical issues were not solved immediately.

The clinician group evaluated the app’s usability in 1-hour sessions in their work environments in every iteration.

Parallel to investigating the usability, feasibility, and safety of the symptom mapping app, the study participants also evaluated a digital solution for biofeedback, as described in the preregistration of the research study Digital Solutions for Concussion [29]. The findings related to the biofeedback device will be reported elsewhere.

### Data Analysis

The outcome measures for each cycle were summarized statistically. Continuous variables were summarized as mean (SD) or median (IQR) according to distribution. Categorical variables were summarized as frequencies (percentages). No statistical hypothesis testing was conducted.

The qualitative interviews were analyzed using reflexive thematic analysis, as described by Braun and Clarke [30], underpinned by a critical realism approach. The first author and fourth author conducted the qualitative interviews, which were later transcribed verbatim by the first author, fourth author, and 2 research assistants. The transcribed interviews were proofread by a research team member other than the transcriber. The first author kept a reflexive journal throughout this process, as well as in the later phases of the analytic process. The first author systematically coded the transcribed data semantically, applying code labels close to the expressed meanings. Next, initial themes were developed by exploring clusters of meaning across the dataset. Through reviewing and further developing the initial themes, the final themes were defined.

The quantitative analyses were performed in SPSS (SPSS Inc) and R (R Development Core Team), and the qualitative analyses were performed in NVivo (Lumivero).

### App Development and Deployment

The app was programmed using React Native and made available for beta testing on both iOS and Android devices. St. Olavs Hospital and Norwegian University of Science and Technology (NTNU) contracted Nordic Brain Tech AS and Fominykh Consulting for consultant services on app programming. During usability testing, the study participants used the app on dedicated study phones provided by St. Olavs and NTNU. During the 28-day home-testing period, the user group used the app on their personal smartphones. They received written instructions and a QR code to download the app, and study staff assisted them by phone. Data collected with the app was transferred as deidentified data to Microsoft through the cloud service Microsoft Azure. The data were encrypted in transit and at rest. The identity and credentials were stored with the identity management service Microsoft Azure AD B2C. St. Olavs Hospital and NTNU have full ownership of the app, all data, images, and results created in the study.

### Ethical Considerations

The study was approved by the Norwegian Medicinal Products Agency (NoMa 22/16250‐7) and the Norwegian Ethics Committees for Clinical Trials on Medicinal Products and Medical Devices (REC-KULMU 422538) and preregistered in ClinicalTrials (NCT05635656). The participants in the clinician group were identified based on their expertise in PPCS and recruited through convenience sampling using professional networks of the authors at Norwegian universities, hospitals, and clinics, aiming to include clinicians of diverse multidisciplinary backgrounds. Recruitment material for the user group was distributed through the hospital, patient organizations, and the Norwegian Centre for Headache Research (NorHead). Interested individuals made contact by email. All participants received oral and written information about the study, and informed written consent was obtained prior to the first study visit. Consent was collected electronically using eFORSK, a web-based application developed and maintained by Central Norway Regional Health Authority’s IT department, Hemit HF. Participants were not compensated for their participation.

## Results

### Overview

A total of 23 adults (median age 52, IQR 34-59 years; 70% females) with PPCS were included in the user group, and 6 clinicians (median age 53, IQR 35-60 years; 50% females) were included in the clinician group. Table 1 displays demographic and injury-related characteristics of the user group. The most common injury mechanism was being struck by or on an object, whereas 5 cases were sport-related. Two participants in the user group dropped out during the study period. Both dropped out before commencing the home-testing period and stated personal reasons not related to the study as the reason for their withdrawals.

The participants in the clinician group (n=6) had a median age of 53 (IQR 35-60), and 50% (3/6) were women. They were medical doctors, specialists in physical medicine and rehabilitation, or clinical neuropsychologists with a mean of 13 (SD 7) years of experience in the field of PPCS.

**Table 1.** Demographic and injury-related variables for study participants in the user group (n=23).

Characteristics		Values
Age at inclusion (years), median (IQR)		52 (59‐34)
Sex, n (%)		
Female		16 (70)
Highest level of completed education, n (%)		
Upper secondary, vocational		5 (22)
Upper secondary, general		4 (17)
Tertiary education		14 (61)
Injury mechanism, n (%)		
Fall		5 (22)
Struck by or on an object		12 (52)
Traffic accident		5 (17)
Violence		1 (4)
Time since injury (years), mean (SD)		5 (4)
History of previous concussions, yes, n (%)		8 (35)
Scores, mean (SD)		
Total score RPQ^a^		35.9 (7.0)
Subscore RPQ somatic		19.4 (3.3)
Subscore RPQ emotional		8 (4.0)
Subscore RPQ cognitive		8.4 (2.4)

a RPQ: Rivermead Postconcussion Symptom Questionnaire.

### App Development

**Figure 2.**
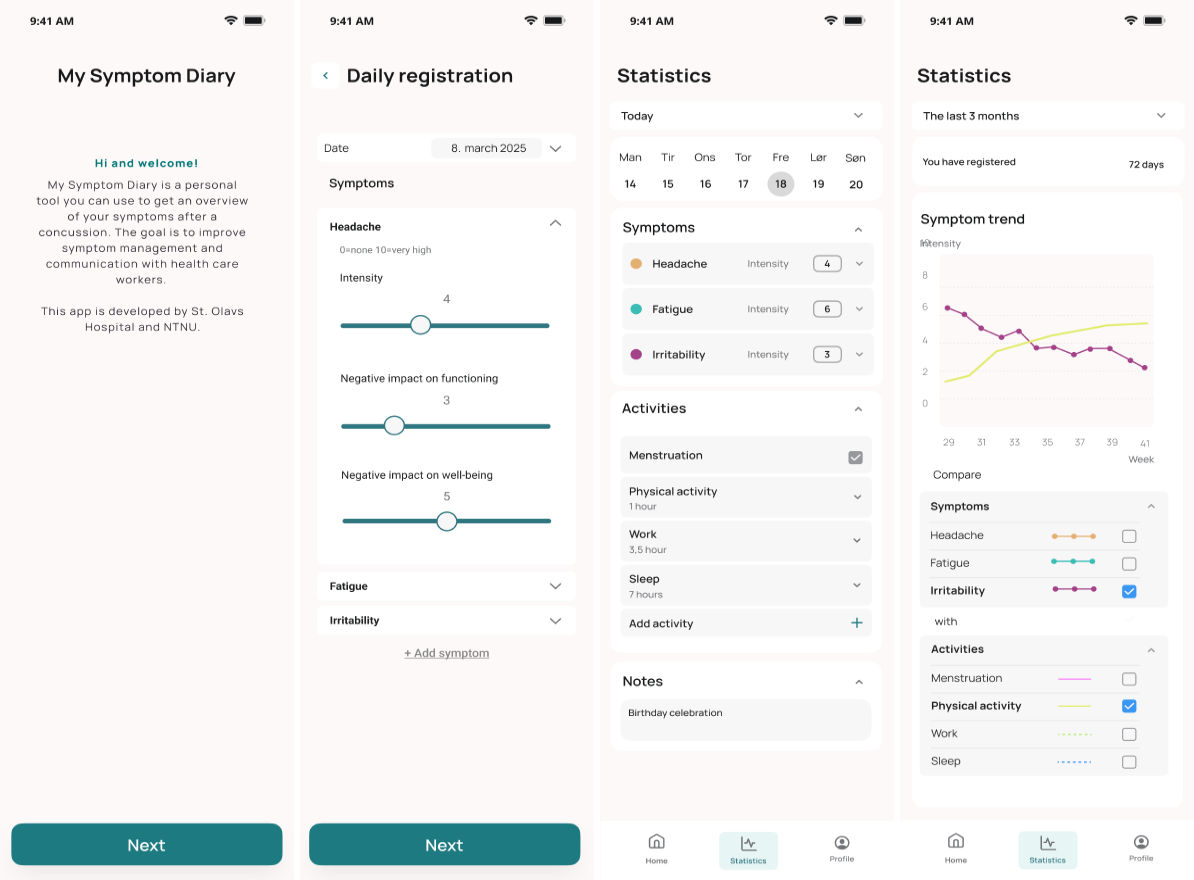
Screenshots from v3 of the app. Screenshot 1 shows the initial screen users see when opening the app for the first time. Screenshot 2 shows the first screen of a diary entry. The drop-down menu for each symptom contains 3 different parameters: intensity of symptoms, negative impact on functioning, and negative impact on well-being. Screenshot 3 shows an overview of the current day. Screenshot 4 shows statistics from the diary entries over the past 3 months in a line graph, with customization options to select which symptoms and activities to display. The study participants used a Norwegian version of the app, but for the purpose of this article, the screenshots have been translated into English.

Three versions of the symptom diary app were developed in the course of the study based on feedback from the study participants during usability testing. The third version of the app was used in the 28-day home-testing by the user group (Figure 2).

### Usability

**Figure 3.**
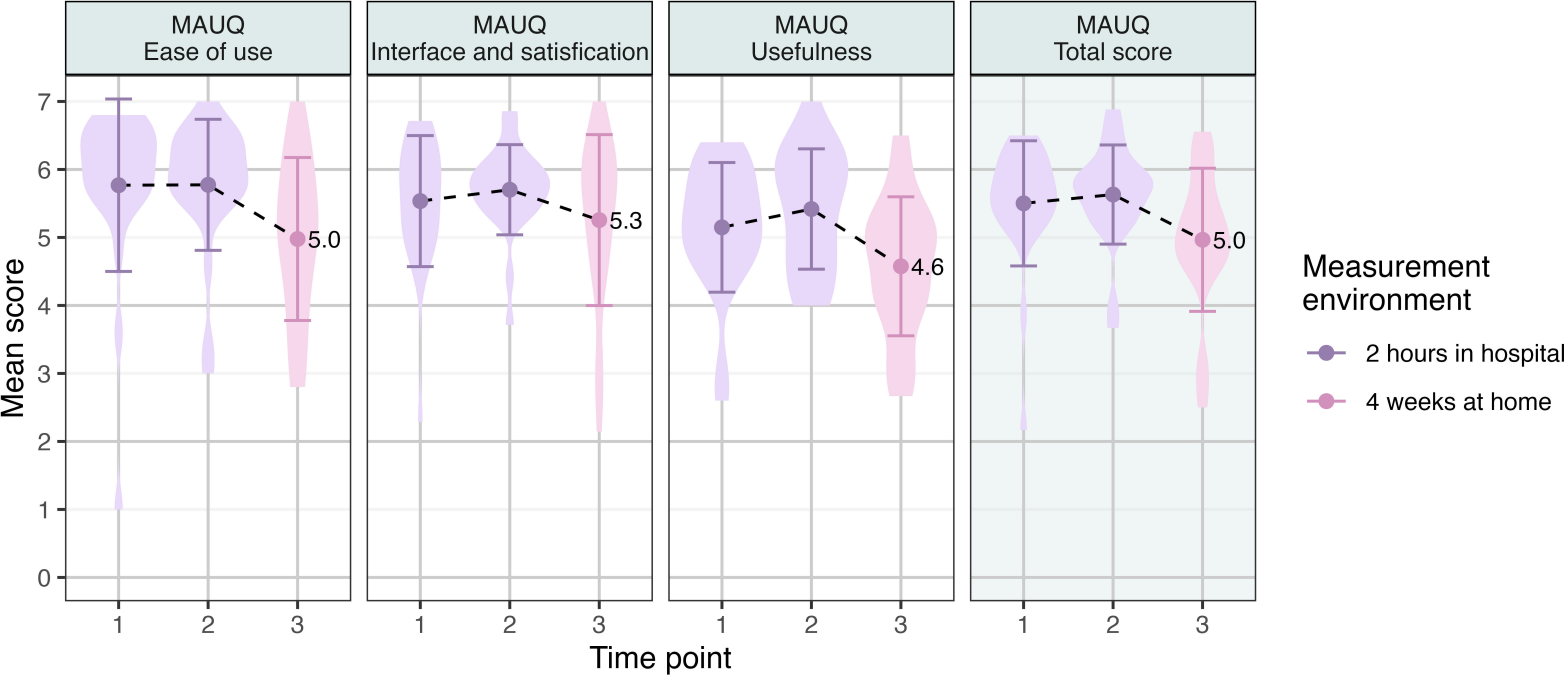
mHealth App Usability Questionnaire scores for the symptom mapping app, completed by the user group. MAUQ: mHealth App Usability Questionnaire.

The user group rated the third version of the app’s usability 5 (SD 1.1) out of 7 on the MAUQ, with the highest scoring for Interface and satisfaction, followed by Ease of use and Usefulness (Figure 3).

**Figure 4.**
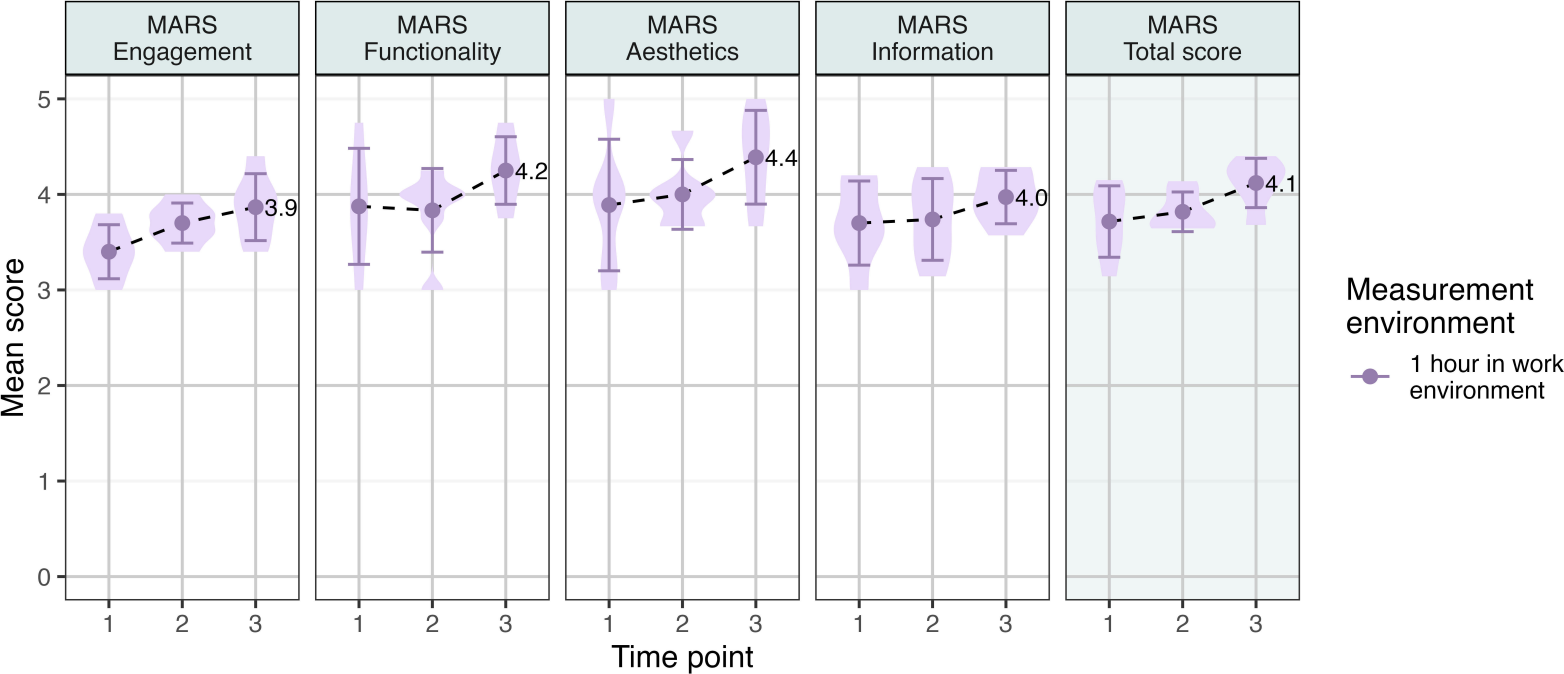
Mobile App Rating Scale scores for the symptom mapping app, completed by the clinician group. MARS: Mobile App Rating Scale.

The clinician group’s mean rating of the app on the MARS was 4.1 (SD 0.4) out of 5, with Aesthetics scoring the highest, followed by Functionality, Engagement, and Information (Figure 4).

### Feasibility

A total of 21 participants in the user group completed the home-testing period, of whom 16 experienced technical issues of different severities during this time. There were 14 participants who experienced mild technical issues, such as being logged out of the app or the app “freezing,” while 2 participants experienced severe technical problems where symptom entries were not saved due to a synchronization issue with the server. Despite the 2 participants’ efforts using the app, significant amounts (possibly >85%) were not stored, and it was therefore not possible to accurately calculate their adherence rate. These were therefore excluded from the adherence analysis. The 2 excluded study participants with severe technical problems successfully completed all other study measures and were not excluded from other analyses. The adherence rate for the user group during the home-testing period was 89% (IQR 78-96), n=19. The adherence rate for all analyzed participants, independent of whether they experienced severe technical issues or not, was 81% (IQR 75-96; n=21).

### Safety

Two AEs were logged during the home testing period. No severe AEs or unexpected severe events were logged. In both incidents, the study participants reported increased awareness of symptoms as an unwanted effect of using the symptom mapping app.

### Qualitative Analysis and Results

Using reflexive thematic analysis through a 6-phase process as described by Braun and Clarke [30], 3 themes were constructed: (1) Visualizing the invisible—Enabling reflection and insight; (2) Personalized yet simple—Balancing relevance and usefulness; and (3) More than just a number—The complexity behind symptom scores.

### Visualizing the Invisible—Enabling Reflection and Insight

The first theme surrounds the participants’ need for a concrete visualization of their experiences with PPCS. They expressed a need to document their symptoms to confirm for themselves that this is actually what it is like over time and not something they are exaggerating or understating. The app also gives the opportunity to engage in self-reflection and identify possible adjustments to improve functioning and well-being. One participant stated:

*It’s about getting an overview of your own life and knowing that, okay, I can see the patterns, on the weeks where I sleep less, it gets worse. And then it’s a bit reassuring to know, okay, I know this will be a hectic period, so maybe I need to take it a bit easier on other things. It’s not necessarily about building your life around this, but it gives you the opportunity to make smarter choices and set yourself up with better conditions.*

[Zara, 64, v3]

The participants described how they can use the visual representations of their reality to take ownership of their condition and proactively manage their own lives. They want to make informed decisions and set priorities that align with their values.

Many also see the potential of sharing such a visual representation of their PPCS experience with health professionals to facilitate communication and create a shared understanding of their symptom burden. One participant explained:

*When you are at the general practitioner’s office, you have very limited time to communicate everything that has been going on for the past two or three months; it is hopeless, actually. The doctor could rather just go in [the app] by themselves, watch the log, and then see how you have been.*

[Omar, 53, v3]

Another participant said:

*When you are in treatment and you get the question ‘how have you been lately?’ it’s not so easy to remember because you are standing so close to it. So, to be able to show your registrations to get it more…in writing, that is usually very helpful*

[Tamara, 56, v1]

They explained that it can be helpful to zoom out and show gradual and nonlinear recovery. Tracking their journey to recovery was thus another potential of the symptom diary pointed out by the participants.

Notably, several participants had already tried to create a visualization of their PPCS experience, using, for instance, Microsoft Excel or Post-It notes. Several participants also mentioned they have searched the internet for an app to keep track of their symptoms. Many had experience with headache diary apps, and some had tried symptom-tracking apps for various chronic illnesses. They had not yet experienced finding an app that took “the whole picture” into consideration, allowing them to track the symptoms and activities perceived as relevant to them. As 1 participant pointed out when talking about the headache diaries:

*I feel like it is not enough to just log the headache. I have several types of headaches, but that is not the biggest challenge in my life. The fatigue, the sleep problems, the periods with bad memory have been far more challenging [...] There is so much more to it (than the headache).*

[Valerie, 47, v1]

### Personalized Yet Simple—Balancing Relevance and Usefulness

This theme represents the fine line between a useful and a cumbersome tool. A useful tool is perceived by the study participants as an app containing the necessary elements to log the aspects relevant to their functioning and well-being, while still being as simple as possible, making it low-threshold to use daily.

Many participants found the app relevant and easy to use, though some found it cumbersome and time-consuming. They expressed a need to minimize the available options and the clicks necessary to complete a symptom registration. They also highlighted the importance of always being able to edit their preselected symptoms.

The symptoms that users wanted to log seemed to be connected not only to symptom intensity, but also to their impact on their functioning, well-being, and symptom fluctuation.

*It can be something that doesn’t occur very often, but it is very intense when it does, or the opposite, something that is present most of the time without being extremely critical, but it’s always there, simmering in the background in a way. So as long as you can choose [...] because people are different*

[Sophie, 25, v2]

The activities perceived as relevant seemed to be connected to their perception of what influences symptom burden, but also activities enhancing their overall well-being without necessarily actively decreasing symptom intensity. For instance, 1 participant said:

*Working in the garden is something that gives me peace of mind, and you need that on a bad day. Yeah, working in the garden would be nice to have as a distinctive activity to register*

[Omar, 53, v3]

Several participants expressed how different contexts could generate different perceptions of what is relevant to track, for instance, starting a new treatment, returning to work after sick leave, or when experiencing a particularly stressful period in their personal lives. The feeling of purpose seemed to be essential for the perception of relevance.

Thus, there was great variation in which symptoms and activities were perceived as relevant. This underlies the importance of a flexible layout allowing for personalization, where the user of the app defines what is relevant in their unique case, enhancing the user’s perception of the app’s usefulness.

### More Than Just a Number—the Complexity Behind Symptom Scores

The third theme reflects the complexity of PPCS and how the mapped symptoms in the app will always remain a simplification of reality. One participant put it this way:

*To me, there is more behind that number than to you, and you can’t include every person’s life story, you need a number, so I get that*

[Pedro, 58, v1]

Many participants found it challenging to sum up their experiences in a score. They acknowledge that there is no correct answer to “what a score of 5 means,” and that they operate the scale based on their perception of what the scores represent, which is not static. One participant explained how they found their scoring became more consistent over time and that they needed about 14 days of “calibration” during the home testing. Another participant highlighted how their perception is, in fact, dynamic:

*If it’s been a long time since you had a 9‐10, and you are in more pain than usual, you might put down a score of 8. But during a bad period, that score of 8 would’ve actually been a 5. So, that is something to keep in mind: a 5 is not always a 5.*

[Eve, 40, v2]

The participants reflected on several aspects complicating their action of scoring, including the frequent fluctuations of symptoms, the intertangled relationship between symptoms, and other factors influencing the experienced symptom burden. Some pointed out internal factors influencing their perception of today’s score, (eg, how they slept), as well as external factors (eg, if they went outside and were exposed to a lot of noise or stayed home that day). One person said they generally hesitated to score a 10 as they felt others probably had it worse.

Some also reflected on what they compared themselves to when scoring, for instance, life before the injury or to their peers.

While recognizing the challenging act of scoring and acknowledging that it inevitably always will be a simplification of their experiences, several participants still eyed the potential benefits of scoring their symptoms:

*It is difficult to register [symptoms] based on a number, but at the same time, it makes it much more measurable and easier to spot patterns*

[Sophie, 25, v1]

## Discussion

### Overview

We developed a research-based mHealth app for symptom mapping for people with PPCS. The app was created by combining rigorous research methods with iterative usability testing, together with people with PPCS and experienced clinicians in the field. We investigated the app’s usability, feasibility, and safety. The app received high usability ratings from both the user and clinician groups and achieved a high adherence rate during home testing. Two AEs related to increased symptom awareness were logged during the study period.

### Usability

The app received generally favorable usability scores and app quality scores from both the user and clinician groups. After 28 days of home testing, the final version received a mean score of 5 (SD 1.1) on the 7-point Likert scale in MAUQ by the user group and 4.1 (SD 0.4) on the 5-point Likert scale in MARS by the clinician group. Previous studies have evaluated apps for tracking symptoms in various conditions, including concussion and chronic pain, using the same questionnaires, with mean MAUQ scores ranging from 4.7‐6.1 [31,32] and mean MARS scores ranging from 1.42‐4.1 [33,34], implying that the app is considered acceptable in regards to usability and app quality by both people with PPCS and clinicians working in the field of PPCS. Notably, the user group rated the first 2 versions of the app higher than the third and final version (Figure 3). The differences in the study setting may partly explain this, as the user group tried the first 2 versions of the app in 2-hour sessions at the hospital, while using the third version at home for 28 days. The participants may have encountered challenges with the app when using it more extensively at home than during the limited time they had at the hospital. Furthermore, many of the participants experienced technical issues in the app during the home-testing period, possibly also affecting their experience of usability. Notably, the completion of the MAUQ did transpire in identical settings at all times, always situated in the hospital.

### Feasibility

The adherence during the 28-day home-testing period was 89% (IQR 78‐96). A meta-analysis of 99 mHealth apps for preventing or managing noncommunicable diseases revealed an average adherence rate of 56% [35]. A study similar to this study developed an app for people in the acute phase of concussions and observed an adherence rate of 28% during a 5-week period [31]. They recruited participants within 14 days of their concussion and thus aimed at a slightly different patient population than in this study, which may partly explain the difference in adherence rate. The high adherence rate in this study implies that the symptom diary app is a feasible approach for logging PPCS despite the relatively high frequency of technical issues for many of the study participants. In this study, we programmed the app to send a daily notification to register symptoms, based on feedback from the user and clinician groups, in order to ensure sufficient data density while also minimizing the experienced burden on the user. Given the high observed adherence rate, future studies can consider exploring multiple registrations per day to enhance data density. However, this should be done while carefully evaluating the user’s experienced burden.

### Safety

Regarding the app’s safety, 2 AEs were registered concerning increased awareness of symptoms during home testing. Both participants completed the home testing but stated during their last encounter with the study personnel that they became more aware of their symptoms, which had a negative impact on their well-being. They both emphasized the value of keeping track of their symptoms but acknowledged that it can also function as a negative reminder. One participant emphasized the importance of receiving guidance from a health care provider in interpreting their symptom logs. This is an important finding, as the effects of symptom tracking in PPCS are understudied. This is, however, not unique to the field of PPCS; the effects of symptom mapping are, in general, understudied. In a small study with healthy individuals, it was shown that subjects tracking their symptoms daily for 2 weeks had increased recall of symptoms and perception of symptom severity compared to the control group [21]. Still, we should not discard the reported experiences of the app’s usefulness despite these incidents. It is plausible that a subgroup of the study population is prone to experiencing unwanted side effects related to increased awareness of symptoms. Future studies should focus on identifying such subgroups and determining which clinical implications symptom tracking apps should have. Developers of mHealth apps or wearable devices measuring symptoms should be aware of their ethical responsibilities, and clinicians recommending such tools and individuals using them should be made aware of the potential side effects [36-38].

### Qualitative Analysis and Results

In the qualitative analysis, 3 overarching themes were created, which together informed the quantitative findings on the apps’ usability, feasibility, and safety. The first theme, Visualizing the invisible—Enabling reflection and insight, actualizes a need for visual representations of PPCS experiences. It is essential for the usefulness of a mHealth app that its purpose is rooted in users’ needs, as a good fit between a mHealth app’s purpose and the user’s needs increases the likelihood of continuous use [39].

The research field of personal visualization offers perspectives on the expressed need for visual representations of PPCS experiences. Visual representations of personal data can empower people with improved access and comprehension of their own data [40]. A systematic review of information visualization found visual tools essential in helping people understand and make sense of their personal health information, which is necessary to improve self-management of their health condition. For chronic illnesses, the “management” is oftentimes not focused on curing the condition but rather on coping and maintaining everyday life [41]. Coping and maintaining everyday life as an overarching goal is in line with the study participants’ reflections on the purpose of the symptom diary app. They describe a need for a tool to visualize their PPCS experiences, create insight, engage in self-reflection, and make informed decisions aligned with their personal values and goals, as well as to facilitate communication with health care providers.

The second theme, Personalized yet simple—Balancing relevance and usefulness, touches upon both aspects of usability and feasibility of the symptom diary app. Experience of relevance seems to be vital to the perception of the app being useful, which again is essential for the probability of a mHealth app actually being used [39,42]. What is perceived as relevant is highly individual, as reflected in this second theme. The diverse views on relevance highlight the importance of a personalized layout in the app, allowing the user to define which symptoms and other variables are relevant to log and visualize in graphics. However, there appears to be a fine line between including many options to ensure perceived relevance and including too many options, making the app overly cumbersome. Thus, this requires careful navigation when developing mHealth apps for people with PPCS.

Finally, the third theme, More than just a number—The complexity behind symptom scores, addresses a more overarching concept, informing all 3 study objectives: usability, feasibility, and safety. Even though most study participants appreciated a visual representation of their PPCS experience and leveraged it to create overview and insight, many acknowledged the challenge of quantifying their experiences. They elaborated on how their interpretation of the scale is dynamic, depending on both internal and external factors. It is an important reminder that the visual representation of a person’s PPCS experience in the app, or any other symptom-tracking instrument, will always remain a simplification of reality. A person’s experience will always be richer and more complex than what can be expressed in numbers and summarized in graphics [43]. However, this does not diminish the usefulness of a visual representation for coping and managing everyday life, as described in the first theme.

Still, an important aspect to consider is how simplifying and quantifying someone’s experience with PPCS may contribute to how they perceive their own experiences. From a critical realist perspective, PPCS exists as a real phenomenon independent of our descriptions of it. However, the conceptual framework embedded in the app, ie, the words we use, the categorization of symptoms, and the operationalization of numerical scales, will to some extent shape how the users of the app perceive, understand, and communicate their experiences. Creating a simplified visual representation can be useful for people with PPCS in gaining an overview and insight into a complex condition, as well as in communicating their PPCS experiences to others. It can support the user of the app in understanding and making sense of their condition, which again is crucial to health management and improving quality of life [41]. Still, this highlights the ethical responsibility of mHealth app developers to create research-based layouts and content [44]. Our findings regarding the symptom diary’s safety illustrate the importance of not taking this ethical responsibility lightly. An mHealth app can have unwanted side effects for some individuals, just as any other health intervention, and requires a research-based approach.

### Limitations

The questionnaires we applied to investigate app usability (MAUQ) and app quality (MARS) have been used extensively and validated in many languages [45,46]. For the purpose of this study, professional translators translated both the MAUQ and the MARS questionnaires into Norwegian using forward-backward translation, followed by harmonization of the translation by experts in the field of PPCS. However, no formal validation of the psychometric properties in a Norwegian context has been done, so direct comparisons of scores in this study with studies using the original English version should be interpreted with caution.

The number of technical issues encountered in the app during the home testing may represent a limitation regarding the feasibility assessment. Yet, adherence was not drastically affected when including the 2 participants who experienced severe technical issues (adherence 89% vs 81%), indicating good adherence despite the technical obstacles. Still, another fourteen participants experienced less severe technical problems, where no data were lost, but it may have affected their daily adherence to the app. Thus, the adherence to the app may be underestimated, but it is, despite this, considered high compared to similar studies investigating the feasibility of mHealth apps in similar study populations [31,35]. The ongoing app development and refinement will further examine technical issues and how to mitigate them in the future.

Finally, in our study sample, 70% were female. Since this study focused on development and usability within a small sample, it was not designed to generalize to the whole PPCS population. Furthermore, women are overrepresented in the PPCS population, with female gender identified as a known risk factor for PPCS [47-49]. Future research should investigate how applicable the findings of this study are to a broader PPCS population.

### Conclusions

The symptom diary app for people with PPCS is considered usable, feasible, and safe for its intended use and is ready for testing in larger-scale clinical trials.

## Abbreviations

AE: adverse event
CENTER-TBI: Collaborative European NeuroTrauma Effectiveness Research in Traumatic Brain Injury
MARS: Mobile App Rating Scale
MAUQ: mHealth App usability questionnaire
mHealth: mobile health
mTBI: mild traumatic brain injury
NTNU: Norwegian University of Science and Technology
PPCS: persisting postconcussion symptoms
RPQ: Rivermead Postconcussion Symptom Questionnaire
